# Laccase immobilization on a natural lignocellulosic biomass support: optimization, characterization, and bisphenol A degradation

**DOI:** 10.1007/s13205-026-04862-4

**Published:** 2026-05-25

**Authors:** Metin Utku Koç, Ceyhun Işık

**Affiliations:** Faculty of Science, Chemistry Department, Muğla Sıtkı Koçman University, Muğla, 48000 Türkiye

**Keywords:** Laccase, Sponge gourd, Lignocellulosic biomass, Immobilization, Bisphenol A degradation

## Abstract

Laccase (LAC) was immobilized onto sponge gourd (*Luffa cylindrica*) through a two step method involving physical adsorption followed by glutaraldehyde-mediated crosslinking. The immobilization parameters were systematically optimized, resulting in maximum catalytic activity (52.98 ± 0.85 U/g) and protein loading efficiency (62.09%) under the following conditions: 2.5 mg/L LAC concentration, 15 mg carrier, 90 min adsorption time, 120 min crosslinking time, and 3% glutaraldehyde. Compared to the free LAC, the immobilized LAC showed a 5 °C increase in optimum temperature and a broader pH activity range. It also exhibited significantly improved thermal and pH stability, retaining 33% and 39% more activity after 240 min at 50 °C and 60 °C, respectively. Storage stability was enhanced, with 57% activity retained after 30 days at 4 °C, compared to only 16% for free LAC. Kinetic analysis revealed a moderate increase in K_m_ (from 0.53 to 0.72 mM) and a decrease in V_max_ (from 0.85 to 0.69 mmol/L min), suggesting minor diffusional resistance. In Bisphenol A (BPA) degradation tests, the immobilized enzyme achieved complete removal of 15 mg/L BPA within 240 min and maintained 46% removal efficiency after 10 reuse cycles. These findings demonstrate that sponge gourd is a cost effective, biodegradable support material that can significantly enhance LAC performance for potential applications in environmental remediation.

## Introduction

BPA is a synthetic compound extensively used in the manufacture of polycarbonate plastics, epoxy resins, and other polymer-based materials. Due to its widespread application in consumer products such as food containers, water bottles, and thermal paper, BPA frequently enters aquatic environments through industrial effluents, leaching, or improper disposal. Its persistence and tendency to bioaccumulate have raised serious environmental and public health concerns (Wang et al. 2022; Neri et al. 2024). BPA is recognized as an endocrine-disrupting chemical, interfering with hormonal signaling and associated with reproductive issues, developmental defects, and increased cancer risks in both humans and wildlife (Akash et al. 2023). Moreover, its chemical stability and resistance to biodegradation render conventional wastewater treatment methods ineffective for complete BPA removal (Abu Hasan et al. 2023; Adamovsky et al. 2024). These challenges highlight the urgent need for sustainable biotechnological approaches that can degrade BPA into less harmful products rather than merely separating it from water.

LACs (EC 1.10.3.2) are multicopper oxidases capable of oxidizing a broad spectrum of phenolic and non-phenolic compounds, with the simultaneous reduction of molecular oxygen to water (Hahn 2023). They are naturally found in fungi, plants, and some bacteria, with white-rot fungi being particularly efficient producers. Due to their broad substrate specificity, ability to function under mild conditions, and use of oxygen as the only electron acceptor, LACs are considered promising biocatalysts for various environmental and industrial applications (Gałązka et al. 2023; Mekureyaw et al. 2025). LACs have found extensive applications in diverse fields, including textile dye decolorization, pulp and paper bleaching, and the degradation of phenolic contaminants and endocrine-disrupting chemicals in wastewater (Younus et al. 2025). However, the practical use of free LAC is limited by its instability under operational conditions such as elevated temperature, extreme pH, or exposure to inhibitors (Scheibel et al. 2024; Zhai et al. 2024). Therefore, improving enzyme robustness and operational stability is crucial for real world applications, particularly in continuous and large-scale bioprocesses (Bolivar et al. 2022).

A widely adopted strategy to enhance enzyme stability and reusability is enzyme immobilization, where enzymes are physically or chemically confined to a solid matrix (Ashkan et al. 2021). Immobilization enhances thermal and pH stability, reduces enzyme loss due to leaching or denaturation, and enables catalyst reuse in batch or continuous systems, which is essential for industrial (Mirsalami et al. 2024; Robescu and Bavaro 2025; Patti et al. 2025). Various immobilization techniques exist, including physical adsorption, covalent attachment, entrapment, and cross-linking, each with advantages and limitations (Holyavka and Artyukhov 2025). While adsorption preserves the enzyme’s conformation, it may result in leaching. In contrast, covalent bonding offers better stability but can negatively impact enzyme activity if not optimized (Tadesse and Liu 2025). Cross-linking often using bifunctional agents like glutaraldehyde can further stabilize the enzyme structure by forming intra- and intermolecular bonds (Robescu and Bavaro 2025). A combined adsorption and crosslinking approach leverages the benefits of both methods, enabling efficient enzyme loading while enhancing structural integrity (Nuraliyah et al. 2021).

The support material plays a crucial role in immobilization efficiency, affecting enzyme activity, loading, and stability (Prabhakar et al. 2025). An ideal support should be mechanically durable, chemically inert or modifiable, biocompatible, and possess a large surface area. In recent years, natural lignocellulosic biomass has attracted attention as an eco-friendly alternative to synthetic carriers. These materials, rich in cellulose, hemicellulose, and lignin, offer abundant functional groups and porous architectures conducive to enzyme binding and mass transfer. Their renewable, biodegradable, and low-cost nature aligns well with the goals of sustainable bioprocesses and green chemistry (Abolore et al. 2024; Muddasar et al. 2024; Kamdem Tamo et al. 2025). One such candidate is sponge gourd (*Luffa cylindrica*), a fibrous biomass with a porous structure, high surface area, and excellent water permeability. These properties support both high enzyme loading and improved substrate accessibility, making it a suitable scaffold for immobilization (Saeed and Iqbal 2013; Małajowicz et al. 2022).

In the context of BPA degradation, LAC offers a unique oxidative mechanism: it catalyzes the one-electron oxidation of phenolic groups in BPA, producing reactive intermediates that undergo coupling or ring-opening reactions, ultimately forming less toxic or mineralized products (Cardullo et al. 2022; Elsayed et al. 2023). Immobilization not only prolongs the enzyme’s activity under such oxidative conditions but also facilitates its reuse and integration into scalable systems such as packed-bed or flow-through bioreactors (Pang et al. 2015; Unuofin et al. 2019; Zhang et al. 2023). Thus, the use of immobilized LAC systems holds strong potential for addressing the limitations of conventional BPA removal strategies.

In this study, a natural sponge gourd support was employed for the immobilization of LAC using a sequential adsorption and crosslinking approach with glutaraldehyde. The immobilization parameters including enzyme concentration, carrier amount, adsorption time, cross-linking time, and cross-linker concentration were systematically optimized. Structural and morphological characterization of the support before and after immobilization was performed using Fourier transform infrared spectroscopy (FTIR) and scanning electron microscopy (SEM) to confirm enzyme attachment and evaluate surface modifications. The performance of free and immobilized LAC was compared in terms of catalytic activity, thermal/pH stability, kinetic parameters, storage stability, and reusability. Finally, the potential application of the immobilized system for BPA degradation was demonstrated through batch removal experiments under optimized conditions.

## Materials and methods

### Materials

LAC (≥ 0.5 U mg^− 1^) from *Trametes versicolor*, glutaraldehyde solution (25%, w/v), 2,2′-azino-bis(3-ethylbenzothiazoline-6-sulfonic acid) (ABTS), BPA (2,2-bis(4-hydroxyphenyl)propane, ≥ 99%), bovine serum albumin (BSA), sodium acetate, potassium dihydrogen phosphate, dipotassium hydrogen phosphate, and trisodium citrate were purchased from Sigma-Aldrich (USA). Sponge gourd (*Luffa cylindrica*), used as the natural lignocellulosic biomass support for enzyme immobilization, was obtained from a local plant market in Muğla, Türkiye. Prior to use, the biomass was washed thoroughly with deionized water and dried at room temperature. All reagents were of analytical grade and used without further purification. Deionized water was used for all solution preparations.

### Preparation of the lignocellulosic carrier

The raw sponge gourd was washed thoroughly with tap water and then with deionized water to remove surface debris. It was then cut into uniform cylindrical pieces (approximately 233 cm in length and 1 cm in diameter) and dried in a hot air oven at 60 °C for 24 h or until constant weight was achieved. The dried material was further chopped into smaller fragments (approx. 0.5–1 cm) to increase surface area. Before use, the fragments were soaked in deionized water for 1 h at room temperature to rehydrate the matrix, drained, and air dried to restore porosity. The prepared carriers were stored in a desiccator (ambient conditions) for up to one week prior to use.

### Immobilization of LAC

LAC was immobilized onto sponge gourd through a two-step process involving physical adsorption followed by glutaraldehyde-mediated covalent crosslinking. Both steps were carried out at 25 ± 1 °C. In the adsorption step, 10–20 mg of sponge gourd pieces (10, 12.5, 15, 17.5, and 20 mg) were incubated with 1 mL of LAC solution at varying concentrations (0.5, 1.0, 1.5, 2.0, 2.5, and 3.0 mg mL^− 1^) in 50 mM sodium acetate buffer (pH 4.5). Adsorption was conducted under static conditions at room temperature for time intervals ranging from 30 to 150 min to evaluate the effect of contact time. After the adsorption phase, glutaraldehyde solution (1–5% v/v) was added to the enzyme-support mixture to induce crosslinking. The crosslinking step was also carried out at room temperature for varying durations (30–150 min) to prevent potential thermal inactivation of the enzyme. After adsorption, freshly prepared glutaraldehyde solution (1–5% v/v) was added directly to the mixture to initiate crosslinking. The reaction proceeded for 30–150 min at room temperature without agitation. After immobilization, the carriers were rinsed with 50 mM sodium acetate buffer (pH 4.5) to remove unbound enzyme and excess crosslinker. Immobilized samples were stored at 4 °C in buffer until use. The immobilization yield (%) was calculated based on the difference in enzymatic activity between the initial LAC solution and the supernatant after immobilization, as described by Eq. (1).

1 $$\:{\rm{Immobilization}}\:{\rm{yield}}\:\left( \% \right) = \:\frac{{{A_{{\rm{initial}}}}\: - \:{A_{{\rm{supernatant}}}}}}{{{A_{{\rm{initial}}}}}}\: \times \:\:100$$

where *A*_initial_​ is the enzymatic activity of the original LAC solution (U mg^− 1^ protein), and *A*_supernatant​_ is the enzymatic activity measured in the supernatant after the immobilization process (U mg^− 1^ protein).

The immobilization capacity (mg protein g^− 1^ carrier), defined as the amount of protein bound per unit mass of support material, was determined based on the protein concentration difference before and after immobilization. Protein content was measured using the Bradford assay, and the value was calculated according to Eq. (2).

2 $$\:{\rm{Immobilization}}\:{\rm{capacity}}\:\left( {\frac{{{\rm{mg}}\:{\rm{protein}}}}{{{\rm{mg}}\:{\rm{support}}}}} \right) = \:\frac{{{P_{{\rm{initial}}}}\: - \:{P_{{\rm{supernatant}}}}}}{{{m_{{\rm{support}}}}}}$$

where *P*_initial_​ and *P*_supernatant​_ denote the total protein content in the initial solution and in the supernatant, respectively (both in mg), and *m*_support​_ is the mass of sponge gourd used (g). All results were expressed as milligrams of protein bound per gram of support.

### LAC activity and protein content assays

The catalytic activity of free LAC was determined spectrophotometrically using ABTS as the chromogenic substrate, following a modified protocol (Luong and Poeaim 2024). The assay mixture contained 900 µL of 50 mM sodium acetate buffer (pH 5.0), 100 µL of 1 mM ABTS solution, and 10 µL of enzyme solution, yielding a total volume of 1.01 mL. Reactions were incubated at 25 °C for 10 min and absorbance was measured at 420 nm using a UV-Vis spectrophotometer with a 1.0 cm path length cuvette. The enzyme activity was calculated using the extinction coefficient of ABTS (ε = 36,000 M^− 1^ cm^− 1^). One unit (U) was defined as the amount of enzyme catalyzing the oxidation of 1 µmol ABTS per minute. For immobilized LAC, 10 mg of enzyme-loaded sponge gourd was incubated with 800 µL of sodium acetate buffer (50 mM, pH 5.0) and 100 µL of 1 mM ABTS at 25 °C for 10 min. After incubation, the carrier was separated by centrifugation at 10,000 rpm for 2 min at room temperature. Absorbance of the resulting supernatant was measured at 420 nm, and the specific activity was calculated using Eq. (3).

3 $$\:Ui\: = \frac{{A\: \times \:1000\: \times \:{V_t}}}{{\varepsilon \:\: \times \:L\: \times \:T\: \times \:m}}$$

where *A* is the absorbance at 420 nm, *V*_t_​ is the total reaction volume (mL), ε is the molar extinction coefficient of ABTS (36,000 M^− 1^ cm^− 1^), *L* is the optical path length (cm), T is the reaction time (min), and m is the mass of the immobilized enzyme preparation (g).

Protein concentrations for both free and immobilized enzyme preparations were determined using the Bradford assay, with BSA as the standard protein (Bradford 1976). For immobilized LAC, the amount of bound protein was estimated by subtracting the protein concentration in the supernatant after immobilization from the initial protein concentration introduced into the system.

Residual activity (%) was defined as the enzyme activity measured after a treatment (e.g., thermal, pH, storage) relative to its initial activity before the treatment. Activity retention (%) refers to the percentage of initial catalytic activity preserved by the immobilized enzyme after repeated use or prolonged storage. For all stability experiments, the initial activity of the untreated enzyme was considered as 100%, and subsequent measurements were expressed relative to this baseline.

### Characterization of lignocellulosic carrier before and after immobilization

Structural and morphological characterization of the sponge gourd support, both before and after LAC immobilization, was performed using FTIR and SEM. FTIR spectra were obtained with a Thermo Nicolet iS10 spectrometer using the KBr pellet method. Approximately 1 mg of dried sample was mixed with 100 mg of spectroscopic-grade KBr, pressed into pellets, and scanned in the 4000 –500 cm^− 1^ range with 4 cm^− 1^ resolution and 32 accumulations per sample. Surface morphology was examined via field-emission SEM (FE-SEM, JEOL JSM-7600 F) at the Advanced Technologies Application and Research Center of Muğla Sıtkı Koçman University. Samples were affixed to aluminum stubs using conductive tape, sputter-coated with a 10 nm gold layer, and imaged at magnifications ranging from 100x to 1000x under an accelerating voltage of 10–15 kV.

### Characterization of free and immobilized LAC

The biochemical characteristics of free and immobilized LAC were assessed via a series of activity and stability assays under controlled conditions. Unless otherwise stated, all activity measurements were performed using the standard ABTS assay described previously: reaction mixtures containing 900 µL of 50 mM sodium acetate buffer (pH 5.0), 100 µL of 1 mM ABTS solution, and 10 µL of enzyme solution were incubated at 25 °C for 10 min, and absorbance was recorded at 420 nm.

The optimum temperature was determined by measuring enzyme activity at various temperatures ranging from 25 °C to 85 °C. Each reaction was performed in triplicate under standard assay conditions. Free and immobilized LAC were incubated at 50 °C and 60 °C for up to 300 min to determined thermal stability. Aliquots were withdrawn at intervals of 30, 60, 120, 180, 240, and 300 min, and residual activity was measured.

The effect of pH on LAC activity was evaluated using different buffer systems (50 mM) across the pH range 2.0–8.0: glycine-HCl (pH 2.0–3.0), sodium acetate (pH 4.0–5.0), sodium phosphate (pH 6.0–7.0), and Tris-HCl (pH 8.0). For pH stability analysis, enzyme solutions were incubated in each buffer at 25 °C for 300 min. Samples were taken at defined time points (0, 60, 120, 180, 240, and 300 min), and residual activity was determined.

Reusability of immobilized LAC was tested over ten consecutive catalytic cycles. After each reaction, the sponge gourd carrier was washed thoroughly with 50 mM sodium acetate buffer (pH 5.0) and reused immediately under the same conditions. Residual activity was determined after each cycle, and results were expressed as a percentage of initial activity.

Enzyme samples were stored at 4 °C and 25 °C in 50 mM sodium acetate buffer (pH 5.0) without substrate to determined the storage stability. Activity measurements were taken every 3 days over a 30-day period to monitor long-term stability.

Kinetic constants (*K*_m_ and *V*_max_) were obtained by measuring initial reaction rates at varying ABTS concentrations (0.05–2.0 mM) under the respective optimal temperature and pH for each enzyme form (35 °C/pH 4.0 for free LAC; 40 °C/pH 5.0 for immobilized LAC). The parameters were calculated by nonlinear regression fitting to the Michaelis-Menten Eq. (4).

4 $$\:\mathrm{V}=\:\frac{{V}_{max}\:\:\left[S\right]}{{K}_{m}\:+\:\left[S\right]}$$

where *V* is the initial reaction rate (U), [S] is the substrate concentration (mM), *V*_max_ is the maximum reaction rate (U), and *K*_m_ is the Michaelis-Menten constant (mM), which represents the substrate concentration at which the reaction rate reaches half of Vmax.

### BPA degradation experiments

BPA concentrations were determined using a colorimetric method adapted from the Standard Methods for the Examination of Water and Wastewater. In this assay, BPA reacts with 4-aminoantipyrine and potassium ferricyanide at pH 7.9 to form a quinone-imine complex with a characteristic absorbance at 507 nm (Lassouane et al. 2019). Absorbance measurements were performed using a UV-Vis spectrophotometer, and BPA concentrations were calculated using a standard calibration curve constructed over the range of 0–25 mg L^-1^ (R^2^ ≈ 0.99).

Comparative degradation experiments were performed using 100 mL of 50 mM sodium acetate buffer (pH 5.0) containing 15 mg L^-1^ BPA in 250 mL Erlenmeyer flasks. All experiments were conducted in the dark (flasks covered with aluminum foil) at 125 rpm. The reactions were carried out under the optimal conditions for each enzyme form: 35 °C/pH 4.0 for free LAC (60 min reaction time) and 40 °C/pH 5.0 for immobilized LAC (240 min reaction time). Aliquots (1 mL) were withdrawn at 0, 30, 60, 120, 180, and 240 min for BPA quantification.

To examine the effect of substrate concentration, experiments were conducted at BPA levels of 5, 10, 15, and 20 mg L^-1^. Reactions were carried out under the optimal pH and temperature conditions of each enzyme. The degradation time was set at 300 min for free LAC and 240 min for immobilized LAC based on their respective degradation kinetics.

Reusability tests of immobilized LAC were performed over consecutive degradation cycles under optimal conditions (15 mg L^-1^ BPA, 240 min, 40 °C, pH 5.0, 125 rpm). After each cycle, the sponge gourd carrier was separated, rinsed thoroughly with 50 mM sodium acetate buffer (pH 5.0), and reused in fresh BPA solution. BPA concentration was measured at the end of each cycle to evaluate catalytic activity retention across repeated uses.

## Results and discussion

### Optimization of immobilization conditions

The immobilization of LAC onto sponge gourd through sequential adsorption and cross-linking was systematically optimized by varying five key parameters: LAC concentration, carrier amount, adsorption time, cross-linking time, and glutaraldehyde concentration. The optimization aimed to maximize both specific activity and protein loading while ensuring the structural stability of the immobilized enzyme. The results for each parameter are discussed below, with emphasis on the mechanistic reasons for the observed trends.

**Fig. 1:**
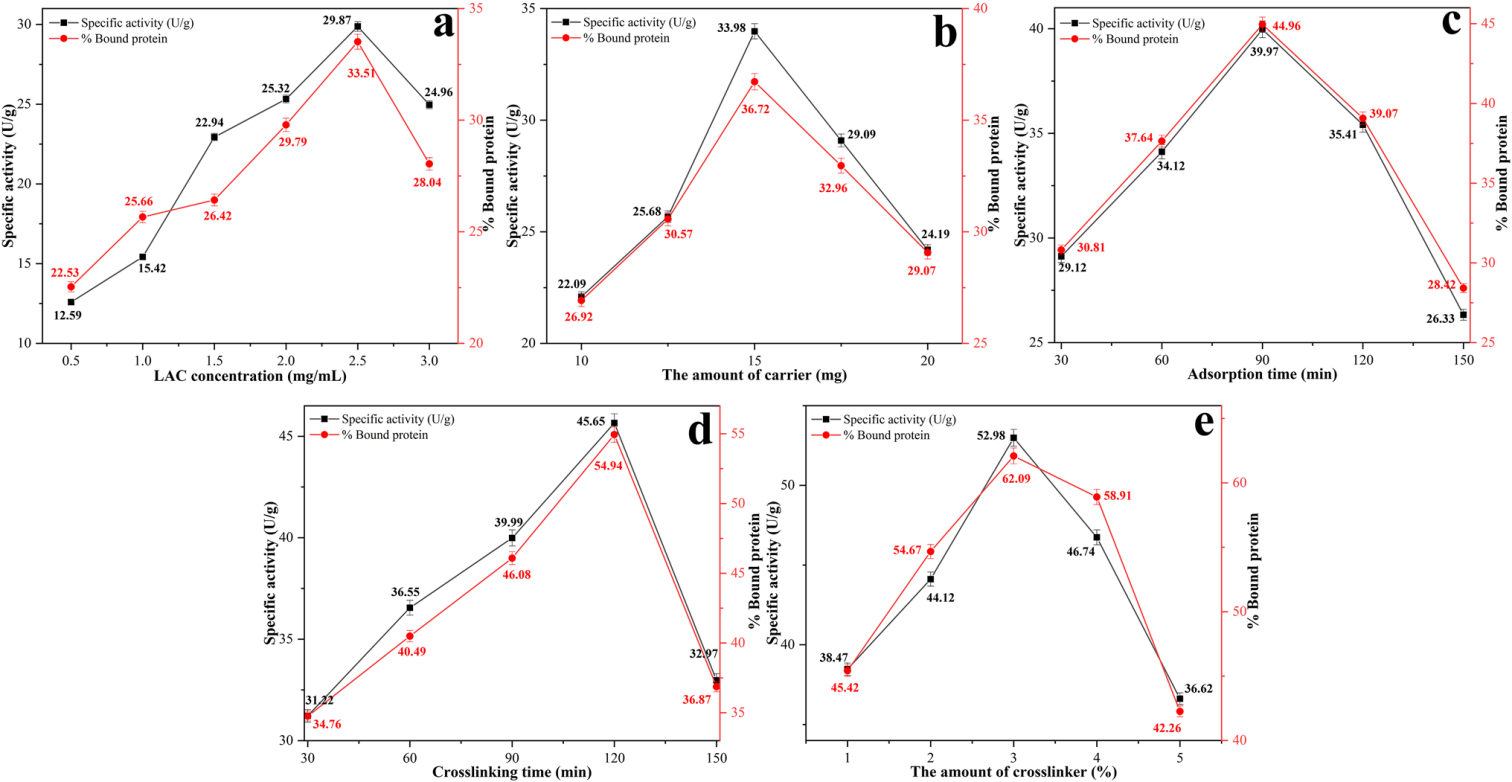
Optimization conditions for LAC immobilization on sponge gourd: **a** enzyme concentration, **b** support amount, **c** adsorption time, **d** crosslinking time, and **e** glutaraldehyde concentration

The effect of initial LAC concentration (0.5–3.0 mg mL^− 1^) on immobilization efficiency is shown in Fig. 1a. Both specific activity and bound protein content increased with increasing enzyme concentration and reached maximum values of 29.87 U g^− 1^ and 33.51%, respectively, at 2.5 mg mL^− 1^. At lower concentrations, the limited enzyme availability resulted in incomplete coverage of the sponge gourd surface and a lower number of accessible active sites, leading to reduced catalytic activity. When the LAC concentration exceeded 2.5 mg mL^− 1^, a decline in both specific activity and protein loading was observed, with values decreasing to 24.96 U g^− 1^ and 28.04% at 3.0 mg mL^− 1^. This decrease can be attributed to overcrowding of enzyme molecules on the carrier surface, which likely caused steric hindrance, restricted substrate diffusion, and partial shielding of active sites. Based on these results, the optimum initial LAC concentration for immobilization on sponge gourd was determined to be 2.5 mg mL^− 1^, providing the highest specific activity and maximum protein binding.

The amount of sponge gourd carrier (10–20 mg) significantly affected the immobilization efficiency of LAC (Fig. 1b). Both specific activity and protein loading increased with increasing carrier amount and reached maximum values of 33.98 U g^− 1^ and 36.72%, respectively, at 15 mg. At lower carrier amounts, the reduced activity was likely due to insufficient surface area for effective enzyme adsorption, resulting in limited enzyme loading and decreased catalytic efficiency. In contrast, when the carrier amount exceeded 15 mg, both specific activity and protein binding declined. At 20 mg, activity dropped to 24.19 U g^− 1^ and protein loading to 29.07%. This decrease may be attributed to a dilution effect, where a fixed amount of enzyme is distributed over a larger surface, leading to reduced enzyme density and limited substrate-enzyme interactions. Based on these findings, 15 mg was selected as the optimal carrier amount for subsequent experiments.

The effect of adsorption time on LAC immobilization was investigated in the range of 30–150 min (Fig. 1c). Both specific activity and protein loading increased progressively with time and reached maximum values of 39.97 U g^− 1^ and 44.96%, respectively, at 90 min. At shorter contact times, the enzyme–carrier interaction had not yet reached equilibrium, resulting in incomplete binding and reduced enzyme loading. At the optimum time of 90 min, equilibrium binding was achieved, allowing for efficient surface coverage and high catalytic activity. Beyond 90 min, a notable decrease in both parameters was observed. At 150 min, specific activity and protein loading dropped to 26.33 U g^− 1^ and 28.42%, respectively. This decline may be attributed to partial desorption of loosely bound enzymes or conformational changes induced by prolonged exposure to the immobilization medium, potentially leading to aggregation and reduced substrate accessibility. Based on these results, 90 min was selected as the optimal adsorption time for further experiments.

The effect of cross-linking time on the immobilization efficiency of LAC was evaluated over a range of 30 to 150 min (Fig. 1d). Both specific activity and protein loading increased with longer cross-linking durations and reached maximum values of 45.65 U g^− 1^ and 54.94% at 120 min. Shorter durations likely resulted in incomplete cross-link formation between glutaraldehyde and the amino groups of adsorbed enzymes, leading to reduced structural stability and partial enzyme leaching. At the optimal cross-linking time, sufficient covalent bonding occurred to ensure enzyme retention while preserving catalytic activity. Beyond 120 min, a marked decrease was observed in both activity and loading, with values dropping to 32.97 U g^− 1^ and 36.87%, respectively. This decline may be attributed to over-cross-linking, which can induce structural rigidity, distort the enzyme’s conformation, and hinder substrate access. Based on these findings, 120 min was selected as the optimal cross-linking duration for immobilization.

The effect of glutaraldehyde concentration (1–5%) on the immobilization efficiency of LAC is presented in Fig. 1e. Both specific activity and protein loading increased with increasing cross-linker concentration and reached maximum values of 52.98 U g^− 1^ and 62.09%, respectively, at 3% glutaraldehyde. At lower concentrations, insufficient covalent bonding between glutaraldehyde and amino groups of the enzyme likely resulted in weak attachment and partial enzyme loss during washing, leading to reduced activity and protein loading. At the optimum concentration, an appropriate balance was achieved between strong covalent fixation and preservation of the native enzyme conformation, resulting in maximum catalytic performance. Further increasing the glutaraldehyde concentration beyond 3% caused a significant decline in both parameters, with values decreasing to 36.62 U g^− 1^ and 42.26% at 5%. This decrease can be attributed to over-cross-linking, which may induce excessive rigidity, conformational distortion of the enzyme structure, and reduced accessibility of the active site, thereby limiting substrate diffusion and catalytic efficiency. Accordingly, 3% glutaraldehyde was selected as the optimal cross-linker concentration for LAC immobilization on sponge gourd.

The optimal conditions for LAC immobilization onto sponge gourd via sequential adsorption and glutaraldehyde cross-linking were determined as follows: enzyme concentration of 2.5 mg mL^− 1^, carrier amount of 15 mg, adsorption time of 90 min, cross-linking time of 120 min, and glutaraldehyde concentration of 3%. These optimized parameters were applied in all subsequent characterization and kinetic experiments.

### Chemical and morphological characterization of lignocellulosic carriers before and after immobilization

**Fig. 2:**
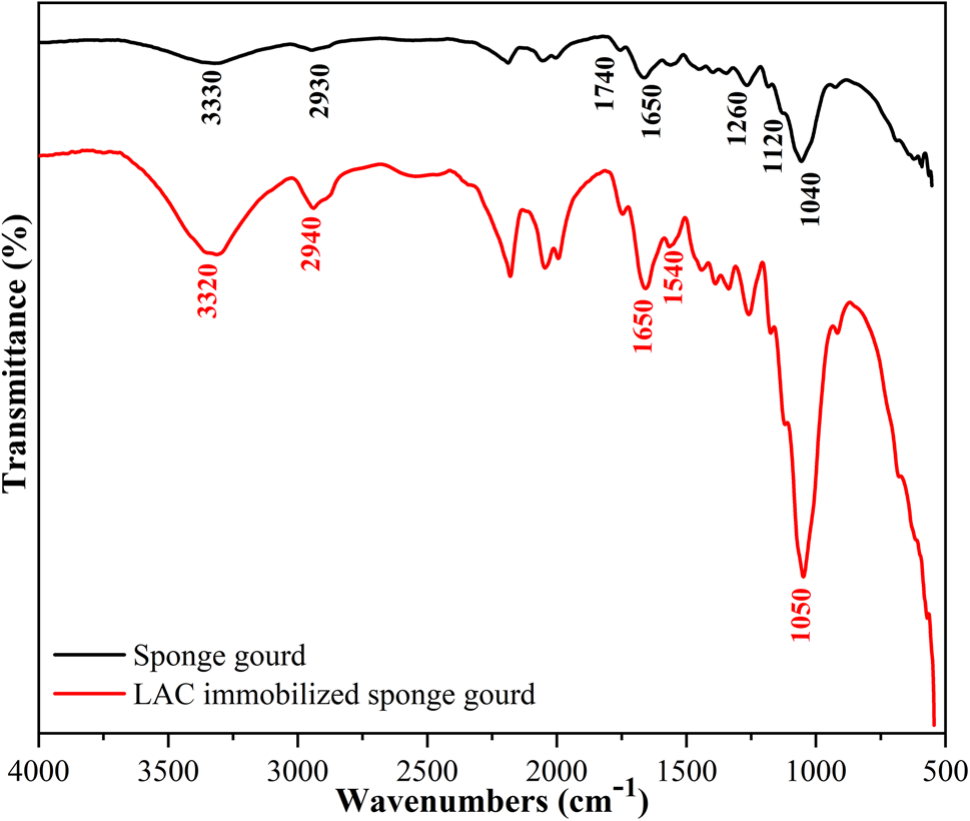
FTIR spectra of sponge gourd and LAC immobilized sponge gourd

The FTIR spectra of raw and LAC-immobilized sponge gourd are shown in Fig. 2. The native sponge gourd exhibited several characteristic absorption bands consistent with the complex lignocellulosic composition of *Luffa cylindrica* fibers, primarily cellulose, hemicellulose, and lignin (Kesraoui et al. 2016). A broad and intense band at 3330–3340 cm^− 1^ corresponds to O–H stretching vibrations arising from hydroxyl groups of lignocellulosic polymers and absorbed water (Kesraoui et al. 2019). Peaks at 2920–2930 cm^− 1^ and 2850 cm^− 1^ are attributed to asymmetric and symmetric stretching vibrations of –CH_2_ groups in the aliphatic cellulose backbone (Schwanninger et al. 2004). The absorption at 1735 cm^− 1^ is assigned to C = O stretching in hemicellulose esters and carboxyl groups, while the band at 1650 cm^− 1^ can be attributed to aromatic C = C stretching of lignin (Tran et al. 2017). Additional features, such as the peak near 1254 cm^− 1^ (hemicellulose and lignin carboxyls) and the bands in the 1119–1035 cm^− 1^ region, correspond to C–O–C stretching and C–O stretching of polysaccharides, respectively (Douissa et al. 2013; Kesraoui et al. 2019). After immobilization, distinct spectral modifications were observed. The broad O–H stretching band became wider and slightly shifted to 3320 cm^− 1^, indicating hydrogen bonding interactions between the hydroxyl groups of the carrier and functional groups of the enzyme. The intensity of the band near 1650–1660 cm^− 1^ increased, consistent with the amide I vibration of peptide bonds, while a new shoulder at 1540 cm^− 1^ appeared, characteristic of the amide II band (N–H bending and C–N stretching), thereby confirming the presence of proteinaceous material on the support. Furthermore, subtle changes in the 1000–1200 cm^− 1^ region suggest the formation of ether linkages, likely arising from glutaraldehyde-mediated crosslinking. Collectively, the appearance of new amide bands and the observed shifts in hydroxyl and carbonyl regions provide clear evidence of successful LAC immobilization onto the sponge gourd matrix.

After immobilization, distinct spectral modifications were observed. The broad O–H stretching band became wider and slightly shifted to 3320 cm^− 1^, indicating hydrogen bonding interactions between the hydroxyl groups of the carrier and functional groups of the enzyme. The intensity of the band near 1650–1660 cm^− 1^ increased, consistent with the amide I vibration of peptide bonds, while a new shoulder at 1540 cm^− 1^ appeared, characteristic of the amide II band (N–H bending and C–N stretching), thereby confirming the presence of proteinaceous material on the support. Furthermore, subtle changes in the 1000–1200 cm^− 1^ region suggest the formation of ether linkages, likely arising from glutaraldehyde-mediated crosslinking. To provide semi‑quantitative support for enzyme immobilization, the relative intensities of the amide I and amide II bands were examined. The clear emergence and increased intensity of these protein‑related bands after immobilization indicate a significant enzyme loading on the sponge gourd surface. The pronounced enhancement of the amide bands provides qualitative and semi‑quantitative evidence for successful LAC attachment. Collectively, the appearance of new amide bands and the observed shifts in hydroxyl and carbonyl regions provide clear evidence of successful LAC immobilization onto the sponge gourd matrix.

**Fig. 3:**
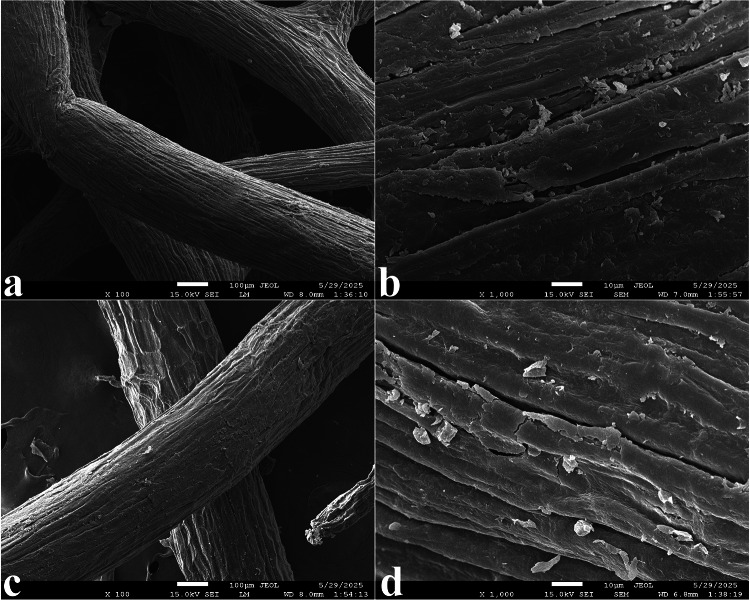
SEM images of the sponge gourd before (**a**, **b**) and after (**c**, **d**) LAC immobilization

The surface morphologies of the native sponge gourd (Fig. 3a and b) and LAC immobilized sponge gourd (Fig. 3c and d) were examined by SEM. The pristine sponge gourd exhibited a well-defined fibrous network structure, with smooth, elongated fibers and distinct grooves along the longitudinal axis. At higher magnification, the fiber surfaces appeared relatively clean, with minimal particulate matter, indicating the absence of significant surface deposits or irregularities. This highly porous and interconnected architecture is advantageous for enzyme immobilization, as it offers a large surface area and open channels that can facilitate mass transfer. Following immobilization noticeable morphological changes were observed. The fiber surfaces became rougher and were covered with irregularly distributed granular and flake-like deposits. These surface features are indicative of the successful attachment of LAC molecules, which may form aggregates or crosslinked networks through the glutaraldehyde-mediated coupling process. In some regions, partial occlusion of the surface grooves was observed, suggesting that the immobilized enzyme layer modified the native porosity of the carrier. Such morphological alterations are consistent with previous reports on enzyme immobilization onto lignocellulosic supports, where protein deposition and crosslinking agents alter the fiber’s microstructure and surface texture (Neto et al. 2023). The observed changes in surface roughness and the presence of particulate layers after immobilization, combined with the FTIR evidence of amide bond formation, provide complementary confirmation of the successful covalent attachment of LAC onto the sponge gourd matrix. This combination of morphological and spectroscopic evidence supports the effectiveness of the adsorption–crosslinking immobilization strategy employed in this study.

### Characterization of free and immobilized LAC

**Fig. 4:**
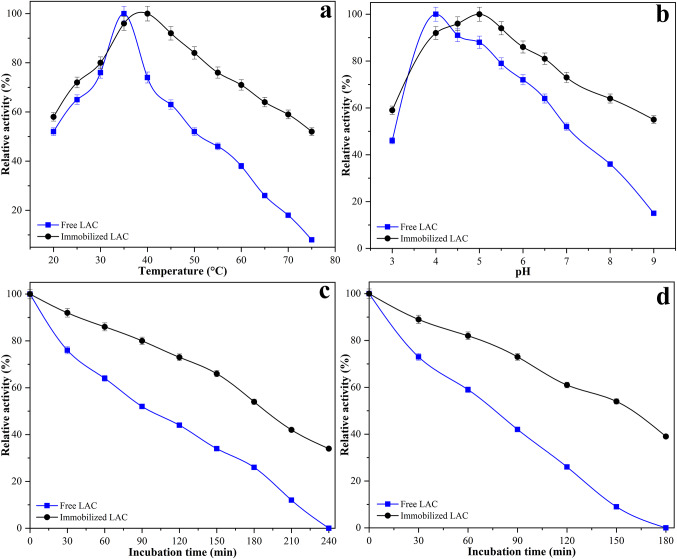
Comparative characterization of free and immobilized LAC: **a** optimum temperature, **b** optimum pH, **c** thermal stability at 50 °C, and **d** thermal stability at 60 °C

The catalytic activity of free and immobilized LAC was evaluated at temperatures ranging from 20 to 75 °C (Fig. 4a). Free LAC exhibited maximum activity at 35 °C, while the immobilized form showed its peak activity at 40 °C, with both forms normalized to 100% relative activity at their respective optima. Below the optimum temperature, both enzyme forms showed increasing activity, consistent with the temperature-dependent enhancement of catalytic rates. Immobilized LAC consistently exhibited slightly higher activity than the free enzyme across the lower temperature range, indicating improved catalytic efficiency under suboptimal conditions. Above the optimum, the activity of free LAC decreased sharply, retaining only 52% at 50 °C and 8% at 75 °C. In contrast, the immobilized enzyme maintained 84% and 52% activity at the same temperatures, respectively. These results indicate that the enhanced performance of immobilized LAC at elevated temperatures is due to structural stabilization, likely achieved by limiting conformational flexibility and minimizing thermal unfolding. The covalent cross-linking step may have further preserved the enzyme’s active site geometry by restricting molecular mobility. The observed shift in optimum temperature from 35 °C for the free enzyme to 40 °C for the immobilized form, along with the significantly higher residual activity at elevated temperatures such as 84% for the immobilized enzyme compared to 52% for the free enzyme at 50 °C, and 52% versus only 8% at 75 °C clearly demonstrates improved thermal tolerance. This highlights the protective effect of the support matrix and covalent cross-linking in maintaining enzyme functionality under thermal stress.

Similar trends have also been observed for LAC immobilized on lignocellulosic-based supports. For example, when luffa sponge was carbonized and magnetized to obtain a magnetic biochar carrier, immobilization of *Bacillus subtilis* LAC resulted in a shift of the optimum temperature from 60 °C for the free enzyme to 70 °C for the immobilized form (Zhang et al. 2020). Likewise, immobilization of *Pleurotus ostreatus* LAC on *Luffa cylindrica* fibers led to an increase in the optimum temperature from 30 °C to 40 °C (Lacerda et al. 2019). These observations support the present results and indicate that immobilization on lignocellulosic matrices, including sponge gourd, can enhance the thermal tolerance of LAC by stabilizing its conformation against heat-induced denaturation.

The catalytic activity of free and immobilized LAC was assessed over a pH range of 3.0 to 9.0 (Fig. 4b). Free LAC exhibited maximum activity at pH 4.0, while the immobilized enzyme reached its peak activity at pH 5.0. Both forms were normalized to 100% relative activity at their respective optima. The shift in optimum pH toward a more neutral value for the immobilized enzyme suggests that the support matrix and cross-linking conditions may create a localized buffering environment, minimizing the impact of proton concentration on the enzyme’s active site. Below the optimum, both forms showed decreased activity; however, immobilized LAC retained higher relative activity across the acidic range. For instance, at pH 3.0, immobilized LAC retained 59% of its activity, compared to 46% for the free form, indicating enhanced acid tolerance. At alkaline pH values, the activity of free LAC declined sharply, retaining only 36% at pH 8.0 and 15% at pH 9.0. In contrast, the immobilized enzyme maintained 64% and 55% activity, respectively, under the same conditions. This improved alkaline stability may be attributed to reduced conformational flexibility, as covalent cross-linking restricts pH-induced structural unfolding. Overall, immobilization shifted the optimum pH from 4.0 to 5.0 and expanded the effective operating range, supporting its suitability for applications in mildly acidic to near-neutral environments.

A similar trend has been observed in previous studies involving lignocellulosic carriers. In the case of *Aspergillus niger* lipase immobilized onto *Luffa cylindrica* sponges, the optimum pH shifted from 7 for the free enzyme to 8 for the immobilized form. Additionally, the immobilized enzyme retained higher activity across the entire pH range tested (2–11) (Zdarta and Jesionowski 2016). This result aligns with the present findings and supports the idea that the lignocellulosic structure of sponge gourd may contribute to the formation of a stabilizing microenvironment around the enzyme, thereby enhancing pH tolerance and extending its effective operational range.

The thermal stability of free and immobilized LAC was evaluated by measuring residual activity after incubation at 50 °C and 60 °C (Fig. 4c and d). At 50 °C, immobilized LAC retained 92% of its initial activity after 30 min, compared to 76% for the free enzyme. After 240 min, the immobilized form preserved 34% activity, while the free enzyme had completely lost its activity. At 60 °C, a similar trend was observed. Immobilized LAC maintained 89% activity after 30 min and 39% after 180 min. In contrast, the free enzyme exhibited 73% activity at 30 min but was fully inactivated by 180 min. These results demonstrate that immobilization significantly improves the thermal stability of LAC. The enhanced resistance to heat-induced inactivation can be attributed to multipoint covalent attachment through glutaraldehyde cross-linking, which likely restricts conformational mobility and helps maintain the enzyme’s active structure. This stabilization effect extends the enzyme’s operational lifetime at elevated temperatures and supports its potential use in processes requiring prolonged thermal exposure.

Comparable findings have been reported in the literature. In a study involving *Trametes versicolor* LAC immobilized on activated carbon derived from green coconut shells, the immobilized enzyme exhibited substantial activity losses, with reductions of approximately 60% and 85% after 2 h at 55 °C and 65 °C, respectively (Mota et al. 2025). These results are in line with the present findings, highlighting that the choice of support material and immobilization method can significantly influence the thermal stability of LAC.

**Fig. 5:**
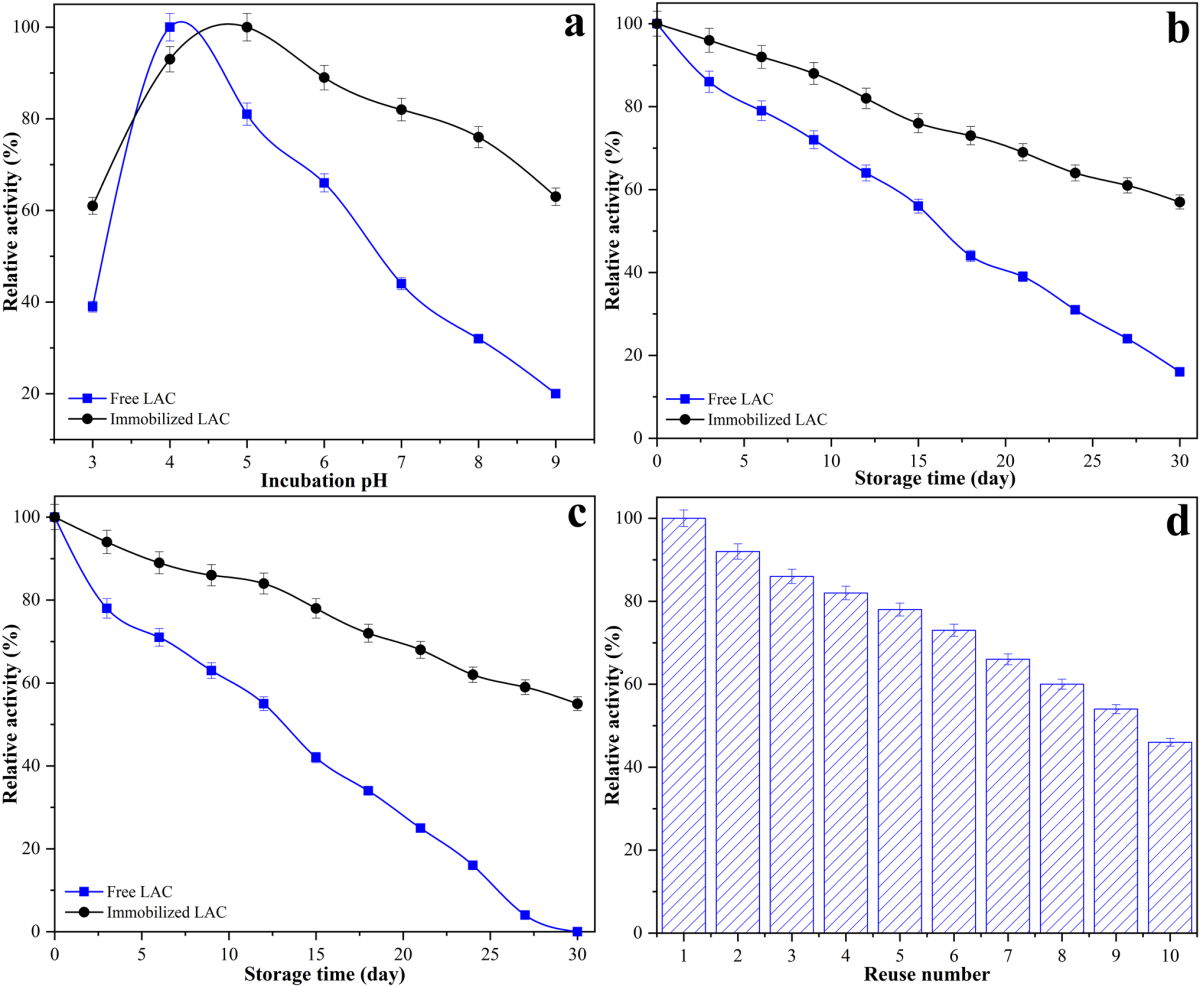
Stability and reusability of free and immobilized LAC: **a** pH stability after incubation at various pH values, **b** storage stability at 4 °C over 30 days, **c** storage stability at 25 °C over 30 days, and **d** operational reusability of immobilized LAC

The pH stability of free and immobilized LAC was evaluated by incubating the enzymes at pH values ranging from 3.0 to 9.0 and measuring the residual activity (Fig. 5a). Free LAC retained maximum activity at pH 4.0, while immobilized LAC exhibited its highest residual activity at pH 5.0. Across the tested pH range, the immobilized form consistently maintained higher relative activity than the free enzyme. For example, at pH 3.0 and 9.0, immobilized LAC retained 61% and 63% of its activity, respectively, compared to 39% and 20% for the free form. This enhanced stability under acidic and alkaline conditions may be attributed to the protective microenvironment provided by the sponge gourd matrix and the covalent attachment via glutaraldehyde, which together reduce conformational flexibility and stabilize the active site. Furthermore, immobilization extended the effective pH range from 4 to 6 (free LAC) to 3–8, improving its applicability under broader operating conditions.

The storage stability of free and immobilized LAC was assessed over 30 days at two temperatures: 4 °C and 25 °C (Fig. 5b and c). At 4 °C, immobilized LAC retained 96% of its initial activity after 3 days and 57% by day 30. In contrast, the free enzyme retained only 86% and 16% activity over the same period. At 25 °C, a more pronounced difference was observed: immobilized LAC preserved 94% activity at day 3 and 55% at day 30, whereas the free enzyme retained 78% activity initially but was completely inactivated by the end of the test period. These findings indicate that the immobilized enzyme has markedly superior storage stability under both refrigerated and ambient conditions. This improvement can be attributed to multiple factors, including enhanced structural rigidity due to glutaraldehyde-mediated cross-linking, which reduces the likelihood of unfolding or denaturation over time. In addition, the lignocellulosic matrix of the sponge gourd support may serve as a protective barrier, reducing exposure to destabilizing environmental factors such as oxidation, moisture fluctuations, and proteolytic attack. Comparing both storage conditions, enzyme inactivation occurred more slowly at 4 °C for both forms, as expected due to reduced thermal and microbial degradation. However, the immobilized enzyme showed a clear advantage even at 25 °C, maintaining over 50% of its initial activity after 30 days. This suggests that immobilization effectively mitigates the destabilizing effects of elevated temperature, which is particularly relevant for industrial settings where cold storage is impractical or cost-prohibitive.

Comparable results have been reported in earlier studies. When *Aspergillus niger* lipase was immobilized on *Luffa cylindrica* sponges, the immobilized enzyme stored at 4 °C retained 95% and 87% of its initial activity after 10 and 20 days, respectively, whereas the free enzyme stored at 20 °C lost nearly 80% of its activity after 20 days (Zdarta and Jesionowski 2016). Similarly, LAC immobilized on bamboo-derived biochar retained as much as 80.1% of its original activity after 30 days at 4 °C, while the free enzyme preserved only 28.14% (Zhang et al. 2025). In another study employing magnetic cellulose composite microspheres, immobilized LAC maintained 75.31% of its activity after 25 days at 4 °C, compared to just 26% for the free enzyme (Yu et al. 2025). These findings are in close agreement with the present work, further confirming that immobilization on lignocellulosic or hybrid supports significantly enhances enzyme longevity under storage conditions.

The reusability of immobilized LAC was assessed over 10 consecutive catalytic cycles under identical reaction conditions, with residual activity measured after each cycle (Fig. 5d). The immobilized enzyme retained 92% of its initial activity after the second cycle, 86% after the third, and 73% after six cycles. By the tenth cycle, 46% of the initial activity was preserved. The gradual decline in activity can be attributed to partial enzyme leaching, mechanical losses during washing steps, and potential structural changes in the enzyme over multiple uses. Nevertheless, maintaining nearly half of the initial activity after ten cycles indicates satisfactory operational stability. The covalent attachment between the enzyme and the support via glutaraldehyde likely reduced desorption, while the sponge gourd matrix provided a stable and reusable carrier. These results suggest that the immobilized enzyme system may be suitable for repeated use in batch processes, offering the potential for reduced enzyme consumption and cost in industrial applications.

### Kinetic parameters

The Michaelis-Menten kinetic parameters of free and immobilized LAC were determined using ABTS as the model substrate (Table 1). The K_m_​​ value for free LAC was 0.53 ± 0.032 mM, while immobilized LAC exhibited a slightly higher K_m_​​ of 0.72 ± 0.042 mM. This increase in the apparent Km following immobilization indicates a reduced effective affinity of the enzyme toward the substrate. Such behavior is commonly attributed to diffusional limitations imposed by the porous structure of the sponge gourd carrier, steric hindrance around the enzyme’s active site, and conformational constraints arising from multipoint covalent attachment via glutaraldehyde. In addition, immobilization may lead to partial shielding of the active site or non-ideal enzyme orientation on the support surface, further limiting substrate accessibility. The maximum reaction velocity (V_max_​) decreased from 0.85 ± 0.028 mmol/L min for free LAC to 0.69 ± 0.054 mmol/L min after immobilization. A reduction in V_max_ is frequently observed in immobilized enzyme systems and is generally associated with restricted molecular mobility, reduced conformational flexibility, and internal mass transfer resistance within the support matrix, which collectively limit catalytic turnover under substrate-saturated conditions. Moreover, it is likely that a fraction of the immobilized enzyme population does not contribute fully to catalysis due to steric constraints or unfavorable orientations. Despite these kinetic changes, the immobilized LAC demonstrated markedly improved thermal, pH, and storage stability, as well as excellent reusability, as discussed in the preceding sections. This behavior reflects a well-recognized trade-off in enzyme immobilization, where modest reductions in apparent catalytic efficiency are often offset by substantial gains in operational stability, longevity, and process robustness key requirements for practical and industrial biocatalytic applications.

Similar trends have been reported in the literature where sponge gourd was used as a natural carrier for enzyme immobilization. For instance, immobilization of *Aspergillus niger* lipase and *Aspergillus niger* pectinase onto sponge gourd supports both led to an increase in K_m_​ and a corresponding decrease in V_max​_ compared to their free forms (Zdarta and Jesionowski 2016; Oluwaseun et al. 2023). These findings are in agreement with the present study, reinforcing that while immobilization on lignocellulosic matrices such as sponge gourd enhances stability and reusability, it often comes at the expense of reduced substrate affinity and catalytic turnover.

**Table 1:** Michaelis-Menten kinetic parameters (K_m_ and V_max_) of free and immobilized LAC

Support	K_m_ (mM)	V_max_ (mmole/L min)
Free LAC	0.53 ± 0.032	0.85 ± 0.028
Immobilized LAC	0.72 ± 0.042	0.69 ± 0.054

### BPA removal experiments

**Fig. 6:**
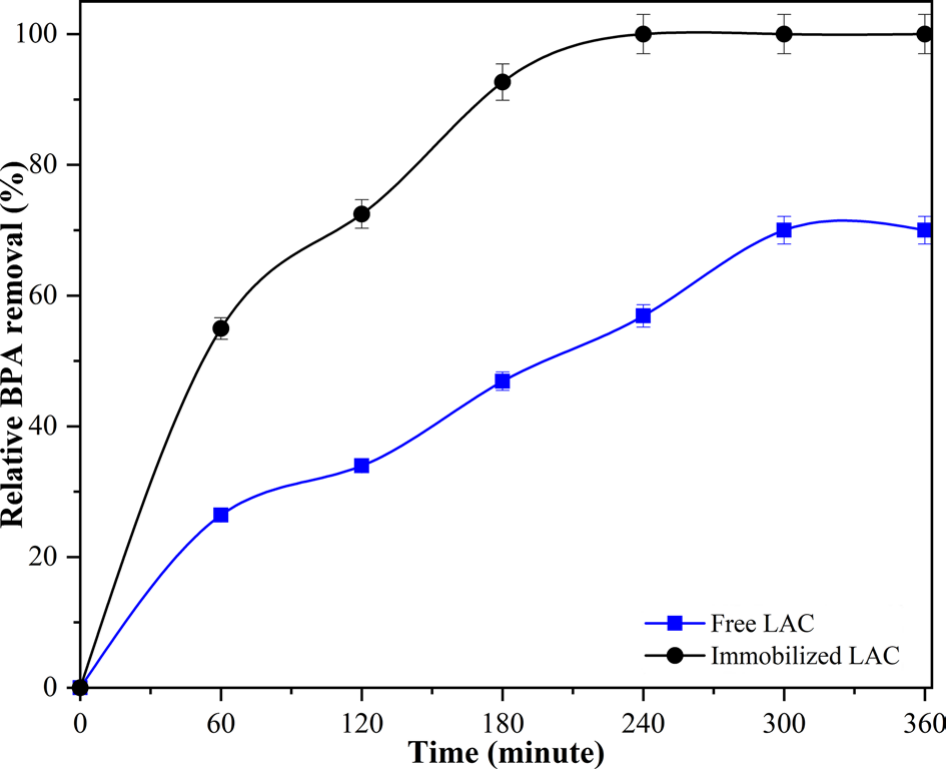
Time dependent BPA removal by free and immobilized LAC under their respective optimal conditions: 35 °C and pH 4.0 for free LAC, and 40 °C and pH 5.0 for immobilized LAC

The BPA removal efficiency of free and immobilized LAC was investigated under their respective optimal temperature and pH conditions, using an initial BPA concentration of 15 mg/L (Fig. 6). Immobilized LAC exhibited a markedly higher removal rate throughout the reaction period compared to the free enzyme. Within the first 60 min, immobilized LAC achieved 54.96% BPA removal, whereas the free enzyme reached only 26.42%. The removal efficiency of immobilized LAC continued to increase rapidly, reaching 92.66% at 180 min and achieving complete BPA removal (100%) by 240 min, which remained constant until the end of the 360-min reaction. In contrast, the free enzyme showed a slower removal profile, reaching 46.89% at 180 min and plateauing at 70% after 300 min. The superior performance of the immobilized enzyme can be attributed to its enhanced stability under the reaction conditions, as confirmed by the thermal and pH stability results, which allow it to maintain higher catalytic activity over prolonged reaction times. Additionally, the adsorption and cross-linking immobilization method likely reduces enzyme inactivation caused by product inhibition or denaturation during the reaction. The faster reaction rate observed for immobilized LAC in the initial phase suggests improved operational robustness and possibly higher effective enzyme concentration due to reduced leaching.

Comparable results have been documented in previous studies. For example, *Myceliophthora thermophila* and *Trametes versicolor* LACs immobilized on polyacrylic films exhibited removal efficiencies of 34.0% and 37.9% after 30 min, 56% and 71% after 1 h, and as high as 91.6% and 93.1% after 2 h at an initial BPA concentration of 80 mg/L (Vallejo et al. 2025). In another report, LAC immobilized within magnetic three-dimensional PEGDA-chitosan inverse opal hydrogels achieved nearly 90% BPA removal within 3 h at 100 mg/L, whereas the free enzyme plateaued at approximately 50% removal after 2 h (Du et al. 2025). These studies, consistent with the present findings, highlight the advantages of immobilization in extending enzyme activity, enhancing catalytic efficiency, and improving the overall removal of BPA from aqueous systems.

**Fig. 7:**
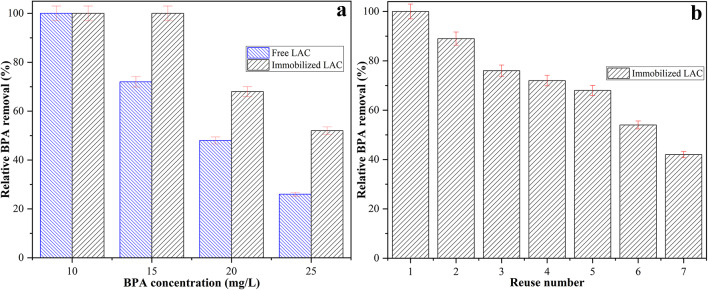
**a** Effect of BPA concentration on the removal efficiency of free and immobilized LAC under optimized conditions, **b** reusability of immobilized LAC for BPA removal over seven consecutive cycles

The effect of initial BPA concentration on removal efficiency was investigated for both free and immobilized LAC under their respective optimal temperature and pH conditions, using reaction times corresponding to the maximum removal observed in the time-course experiments (300 min for free LAC and 240 min for immobilized LAC) (Fig. 7a). At an initial BPA concentration of 10 mg/L, both enzyme forms achieved complete removal (100%). However, as the initial concentration increased, removal efficiency declined for both, with immobilized LAC consistently outperforming the free enzyme. At 15 mg/L, immobilized LAC still achieved complete removal (100%), while free LAC removed only 72% of BPA. At 20 mg/L, removal dropped to 48% for the free enzyme but remained higher at 68% for the immobilized form. At the highest concentration tested (25 mg/L), immobilized LAC removed 52% of BPA, compared to only 26% for free LAC. The decline in removal efficiency at higher BPA concentrations can be attributed to substrate inhibition effects, in which excessive BPA molecules compete for access to the enzyme’s active site, potentially leading to steric hindrance or the formation of non-productive enzyme–substrate complexes. Additionally, the accumulation of reaction intermediates and products at higher pollutant loads may contribute to partial enzyme inactivation. The consistently superior performance of immobilized LAC across all tested concentrations reflects its greater stability and resistance to such inhibitory effects, likely due to the structural rigidity and protection afforded by the sponge gourd matrix and multipoint covalent attachment. These results demonstrate that immobilized LAC maintains high removal efficiency even under elevated pollutant concentrations, which is advantageous for treating real wastewater streams where contaminant levels can vary widely. The capacity to sustain complete or near-complete removal at moderate concentrations further underscores its potential for practical environmental remediation applications.

Comparable results have been reported in previous studies. In one work, LAC immobilized onto MIL–100(Fe)@chitosan nanofiber composites achieved removal efficiencies of 100%, 96%, 91%, 90%, and 83% at BPA concentrations of 1, 2, 5, 10, and 20 mg/L, respectively, with decreasing efficiency at higher concentrations due to saturation effects and substrate competition (Kiaee Nasab Abkenar and Dehnavi 2025). Similarly, in another study utilizing magnetic cellulose composite microspheres as supports, BPA degradation decreased markedly with increasing concentration: approximately 80% removal was achieved at 40 mg/L, whereas only about 40% removal was observed at 120 mg/L (Yu et al. 2025). These findings align closely with the present results, reinforcing that while immobilization significantly enhances enzymatic degradation performance, substrate concentration remains a critical limiting factor due to active site saturation and diffusional barriers.

The operational stability of immobilized LAC was further evaluated through consecutive batch BPA removal cycles performed under optimized reaction conditions (Fig. 7b). The immobilized enzyme retained 89% of its initial BPA removal efficiency after the second cycle, followed by a gradual decline to 76% after the third, 68% after the fifth, and 42% after the seventh reuse. The observed decrease in performance over repeated cycles is likely due to several factors, including partial enzyme leaching from the support, conformational changes in the enzyme’s active site due to repeated substrate and product interactions, and possible fouling or blockage of active sites by BPA oxidation products or polymeric byproducts. Nonetheless, the immobilized LAC maintained over 70% removal efficiency for up to four consecutive cycles, highlighting its robustness for repeated use in pollutant degradation processes. These results reinforce the operational advantages of immobilized biocatalysts in practical water treatment applications, where the ability to reuse the same catalytic batch over multiple cycles can significantly reduce operational costs and enhance process sustainability.

Comparable trends have been documented in earlier reports. For instance, LAC immobilized on MIL–100(Fe)@chitosan nanocomposites exhibited high initial BPA removal efficiencies (100%, 96%, 92%, 85%, and 81% at 1–20 mg/L, respectively), but after five cycles, the efficiencies dropped to 94%, 85%, 71%, 58%, and 42%, with stronger declines at lower BPA concentrations due to accumulation of enzyme–radical complexes (Kiaee Nasab Abkenar and Dehnavi 2025). Similarly, in a study employing rGO-LA sponges, the immobilized enzyme preserved only 57% of its initial activity and 52% BPA removal efficiency after five cycles, attributed to incomplete regeneration, fouling, and gradual enzyme inactivation (Moayedi and Yousefi 2025). Another investigation utilizing Lac-MC@Fe_3_O_4_ composites reported over 50% retention of removal activity after five reuse cycles and > 35% after eight cycles, although activity eventually declined to 10.5% after ten cycles, primarily due to enzyme leaching under mechanical stirring (Yu et al. 2025).

Taken together, these findings are consistent with previous reports and confirm that while immobilization significantly enhances the operational stability and reusability of LAC for BPA degradation, a gradual performance loss over extended reuse cycles is generally unavoidable due to enzyme inactivation, leaching, and surface fouling by reaction intermediates. Nevertheless, the retention of a substantial fraction of catalytic activity across multiple cycles highlights the strong potential of immobilized LAC systems for practical wastewater treatment applications. Although the specific degradation intermediates were not identified in the present study, the observed BPA removal behavior is consistent with the well established LAC mediated oxidative degradation mechanism reported in the literature. LAC typically initiates BPA transformation through one-electron oxidation of phenolic hydroxyl groups, generating phenoxy radical species. These reactive intermediates can subsequently undergo radical coupling, molecular rearrangements, ring opening reactions, or quinone formation, ultimately leading to lower molecular weight products with reduced estrogenic activity. In immobilized systems, the confined microenvironment of the support matrix may further facilitate sustained radical mediated oxidation by stabilizing reactive intermediates, limiting enzyme deactivation, and prolonging effective enzyme substrate contact. This mechanistic framework provides a plausible explanation for the enhanced and sustained BPA removal observed with immobilized LAC in the present study.

## Conclusion

In this study, LAC was successfully immobilized onto sponge gourd via a combined adsorption and glutaraldehyde-mediated crosslinking approach, and the process was systematically optimized. The optimal immobilization conditions were determined as 2.5 mg/L enzyme concentration, 90 min adsorption, 120 min crosslinking, and 3% (v/v) glutaraldehyde, resulting in a maximum activity of 52.98 ± 0.85 U/g and a protein loading efficiency of 62.09%. Compared to the free enzyme, the immobilized LAC exhibited an increased optimum temperature (from 35 °C to 40 °C) and broader pH tolerance, along with significantly enhanced thermal and pH stability. At 50 °C and 60 °C, the immobilized enzyme retained 33% and 39% more activity, respectively, after 240 min of incubation than the free counterpart. Storage stability was also markedly improved, with 57% activity retained after 30 days at 4 °C, compared to only 16% for the free enzyme. Kinetic analysis revealed a slight increase in *K*_m_ (0.53 to 0.72 mM) and a moderate decrease in *V*_max_ (0.85 to 0.69 mmol L⁻¹ min⁻¹), indicating minor diffusional limitations while preserving catalytic efficiency. Application studies demonstrated complete removal (100%) of BPA at 15 mg/L within 240 min under optimal conditions, whereas the free enzyme achieved only 70% removal within 300 min. The immobilized enzyme maintained 46% BPA removal efficiency after 10 consecutive reuse cycles. These results confirm that sponge gourd is an eco-friendly, low-cost, and efficient carrier for LAC immobilization, offering improved stability, reusability, and pollutant degradation performance, making it a promising candidate for industrial and environmental bioremediation applications.

## Data Availability

Not applicable.
